# Modeling Human Ductal Carcinoma In Situ in the Mouse

**DOI:** 10.1007/s10911-018-9408-0

**Published:** 2018-08-25

**Authors:** Fariba Behbod, Angelica M. Gomes, Heather L. Machado

**Affiliations:** 1 Department of Pathology and Laboratory Medicine, University of Kansas Medical Center, Kansas City, KS USA; 2 Department of Biochemistry and Molecular Biology, Tulane Cancer Center, Tulane University School of Medicine, 1430 Tulane Ave, #8543, New Orleans, LA USA

**Keywords:** Ductal carcinoma in situ, Premalignancy, Mammary intraepithelial neoplasia

## Abstract

Breast cancer development is a multi-step process in which genetic and molecular heterogeneity occurs at multiple stages. Ductal carcinoma arises from pre-invasive lesions such as atypical ductal hyperplasia (ADH) and ductal carcinoma in situ (DCIS), which progress to invasive and metastatic cancer. The feasibility of obtaining tissue samples from all stages of progression from the same patient is low, and thus molecular studies dissecting the mechanisms that mediate the transition from pre-invasive DCIS to invasive carcinoma have been hampered. In the past 25 years, numerous mouse models have been developed that partly recapitulate the histological and biological properties of early stage lesions. In this review, we discuss in vivo model systems of breast cancer progression from syngeneic mouse models to human xenografts, with particular focus on how accurately these models mimic human disease.

## Introduction

Human ductal carcinoma in situ (DCIS) is characterized by malignant epithelial cells confined to the milk ducts of the breast, with no evidence of invasion through the basement membrane. It is the most common form of pre-invasive breast cancer, accounting for 20–25% of all newly diagnosed breast cancers and about 60,000 U.S. cases diagnosed every year [1]. Originally proposed by Wellings and colleagues, it is currently well-accepted that ductal cancers originate as atypia, progress to atypical ductal hyperplasia (ADH; premalignant) and ductal carcinoma in situ (DCIS; pre-invasive), the latter of which is a non-obligate precursor of invasive ductal carcinoma (IDC; invasive) and metastatic cancer (metastatic) [2–4]. Similarly, lobular cancers are believed to progress from atypical lobular hyperplasia (ALH) and lobular carcinoma in situ (LCIS). The remainder of this review will focus on ductal carcinomas and in vivo models that mimic DCIS progression to IDC.

DCIS can also be classified histologically according to lesion size, cytonuclear atypia and degree of necrosis. Based on these parameters, DCIS can be classified into low, intermediate and high-grade. Low and intermediate grade DCIS can present with different architectural patterns into comedo, cribriform, papillary, micropapillary, and solid subtypes [5]. Recently, there has been a significant increase in the rate of DCIS detection from 5.8 per 100,000 women in the 1970s to a plateau of 32.5 per 100,000 women since 2004 [6, 7], while, the rate at which breast cancer patients present with late stage disease has only decreased by 8% [8]. Although it is a precursor to invasive cancer, it is estimated that as few as 25% (~43% average) of DCIS patients left untreated will develop invasive disease [9]. However, due to our inability to distinguish lesions that will progress to invasive cancer from those that will remain non-invasive indefinitely, all DCIS patients are treated with surgery and/or radiation. This overtreatment dilemma has sparked controversy amongst physicians and breast cancer patients, posing the questions: *Should all DCIS patients be treated? How should we treat DCIS?* Thus, there remains an unmet need to develop molecular-based approaches to more accurately predict disease progression and overall patient outcome.

Molecular and cellular mechanisms underlying the progression of DCIS to invasive breast carcinoma remains largely unknown. DCIS possess similar inter- and intra-tumoral heterogeneity as invasive breast cancers. In fact, the intrinsic subtypes of luminal, basal and HER2 overexpressing, also exist in DCIS [10]. Similarly, immunohistochemical analysis of DCIS show expression of multiple histologic grades as well as different levels of biomarker expression, including ER, PR, HER2 and Ki67, within the same patient DCIS suggesting that DCIS exhibit similar intra-tumoral heterogeneity as IDC. Indeed, there was a significant correlation between a mutation in p53 and DCIS intratumoral heterogeneity. Based on these data, it is postulated that poorly differentiated DCIS may evolve from well-differentiated DCIS by gradual acquisition of genetic instability imposed by mutated p53 [11].

Traditionally, molecular studies of DCIS progression have been hindered due to limited model systems that recapitulate the molecular and genetic heterogeneity of DCIS. Additionally, few transgenic mouse models progress through distinct stages of premalignancy, such as atypia, ADH and DCIS. In this review, we discuss the advantages and limitations of numerous syngeneic mouse and human-in-mouse xenograft models that are commonly used and most accurately mirror the transition from DCIS to invasive breast cancer.

## Premalignant Lesions in the Mouse: a Historical Perspective

More than 150 years ago, the first scientific observation of a mouse mammary tumor was made [12], yet prevention and treatment schemes of human breast cancer remain a challenge. In the early 1900s, Apolant and Halland described that mouse mammary tumors were of epithelial origin, rather than from connective tissue as believed, and progressed through different stages [13, 14]. In 1938, Fekete and colleagues observed that some mouse mammary hyperplastic lesions, but not all, progressed to invasive tumors [15]. Subsequently, Gardner shed light on the complexity of premalignancy when he showed that hyperplasias were either ductal-derived or alveolar-derived.

In the 1950s, pioneering studies from DeOme and colleagues laid the foundation for using transplantation techniques to study mammary tumorigenesis. They demonstrated that the mammary epithelial ductal tree could be surgically removed from a 3-week-old female mouse, leaving an epithelial-free (“cleared”) mammary gland. As a result, mammary tissue could be transplanted into the “cleared” mammary fat pad, where proliferation and differentiation occurred allowing complete reconstitution of the mammary gland [16]. DeOme showed that upon serial transplantation, hyperplastic lesions recapitulated their previous phenotype. Furthermore, it was observed that hyperplastic lesions were direct precursors of aggressive mammary tumors. Seminal studies from Medina and co-workers showed that hyperplastic alveolar nodules (HAN) transplanted into the cleared mammary gland could expand and fill the fat pad, however, when transplanted subcutaneously, these lesions were viable but could not grow. In contrast, transplantation of tumor cells into any site resulted in tumor development and consequent metastasis. Another interesting feature that distinguished HAN from tumors is that when transplanted into a mammary gland, HAN cannot grow in the presence of endogenous mammary epithelium [17]. Finally, Daniel and colleagues showed that upon serial transplantation, normal mammary tissue had a finite lifespan and initiated a senescence program after 6–7 generations [18], while hyperplastic lesions were immortal [19].

The methodology of mammary transplantation opened new doors, allowing for introduction of normal, premalignant and malignant cells into cleared hosts. As a result, the currently accepted concept of multistage carcinogenesis was proposed in 1967. DeOme suggested that normal cells could develop into hyperplasias [20], and subsequent studies by Medina and others suggested that hyperplastic lesions had an increased potential to become cancerous lesions as compared to normal mammary epithelial cells [21]. Additional studies showed that other stimuli, such as hormones, viruses or carcinogens could stimulate hyperplastic progression [22, 23]. The multistage model of mammary tumorigenesis is now well-accepted in which a linear and branched progression from normal to hyperplasia to neoplasia occurs.

## Mouse Models of Early Stage Progression

### Mammary Intraepithelial Neoplasia (MIN) as a Model of Human Breast Premalignancy

As many mouse models of mammary tumorigenesis are stochastic, appropriate models that recapitulate the progression of early stage lesions has been challenging. In 1999, a panel of expert pathologists (“The Annapolis Pathology Panel”) were asked by the NIH Breast Cancer Think Tank to develop a classification for early stage lesions in genetically engineered mouse models (GEMMs) [24]. The consensus report recommended that hyperplasias associated with cellular atypia with the potential to progress to invasive cancer be referred to as mammary intraepithelial neoplasia (MIN). To date, MIN lesions have been observed in mice expressing endogenous mouse mammary tumor virus (MMTV), chemically-induced carcinomas, and various GEMMs. The panel also recommended that all MIN models should have features of focality, atypia, association with a known cancer, and validation. Focality refers to the appearance of a subpopulation of cells distinct from hyperplastic cells with dense cellularity and atypical cytology. Atypia consists of lesions that comprise cells with enlarged nuclei showing variations in size and shape (pleomorphism), hyperchromatic nuclei (dark stained nuclei; increased DNA content) and abnormal chromatin patterns. There should also be an abnormal proliferation rate when compared to wildtype proliferating pre-lactating mammary glands. Association refers to lesions that have demonstrated an association with a known neoplasm. Association is best represented when a lesion is in direct continuity with an identifiable malignancy, or when a cytological continuum exists between early lesions and invasive cancer. It was also suggested that the lesions are assigned a grading as high vs. low grade. Low grade lesions consist of alveoli or ducts with one or two layers of atypical luminal epithelium. Notably, the majority of GEMM-induced MIN have multi-layered epithelium and are thus considered high grade. Finally, validation of MIN should be performed by transplantation. Hyperplastic lesions found in the mouse mammary gland should be surgically removed and transplanted into the cleared mammary fat pads of syngeneic recipient mice to validate neoplastic potential. Although these guidelines were written almost 20 years ago, they remain the standard for classification of early stage lesions in the mouse mammary gland.

### HAN and DH

Mouse models of early stage progression generate distinct morphological types of MIN including hyperplastic alveolar nodules (HAN), ductal hyperplasia (DH), and cystic lesions. While cystic lesions are benign, HAN and DH have been shown to be precursors of invasive lesions and as such may appropriately represent human DCIS [25]. HAN can be induced by endogenous and exogenous tumor viruses [16, 26], chemical carcinogens [27], irradiation [22], and prolonged hormone stimulation [23]. HAN are an expansion of individual alveolar units that are morphologically analogous to the hyperplastic enlarged lobular units (HELU) seen in early stage breast lesions that progress to ADH and DCIS [4, 28]. However, unlike human premalignant lesions, HAN most often lack hormone receptors such as the estrogen receptor (ER) and are ovarian-independent for growth [29]. Interestingly, these hyperplasias are dependent on the mammary stroma for proliferation and neoplastic transformation. One of the earliest observations of HAN occurred in MMTV-infected mice [Nusse and Varmus 1982]. The MMTV-associated integration sites identified in invasive mammary tumors, including *wnt-1*, *int-2* (*fgf3*), *int-3* (*notch4*) and *int-6* (*elf3e*), have been found in MMTV-induced HAN [30–32]. Serial transplantation of MMTV-induced HAN showed that any given stage of hyperplasia could progress to invasive cancer at various rates. Multiple growth pathways are misregulated, with defects in cell cycle regulation and subsequent genetic alterations [33]. Although initial studies of MMTV-infected mice contributed greatly to our understanding of HAN progression, this model is rarely used to study the biological mechanisms that regulate DCIS progression. Rather, the discovery that the MMTV-LTR (long terminal repeat) is hormonally regulated rendered this viral promoter a useful genetic tool to drive expression of genes in the mouse mammary gland [34].

DH is a second type of MIN that can be induced by chemical carcinogens [35], irradiation [36], progestins [37], and some *TP53*-null GEMMs [38–40]. DH are characterized by an increase in the number of small ducts and/or an increase in the number of epithelial cell layers within a duct [21]. Since DH undergo intraductal epithelial proliferation, these lesions recapitulate many features of human DCIS. Similar to HAN, DH show immortality, association with invasive cancers, and site dependence. In chemical carcinogen-induced lesions, DH appear earlier than HAN. DH induced by some transgenic and chemically-induced models exhibit a higher frequency of hormone-dependence and genetic instability, and thus may mimic the histologic, biologic and genetic features seen in human premalignant lesions more faithfully than HAN [21].

### GEMMs

Over 25 GEMMs of breast cancer have been reported to show evidence of hyperplasia, and molecular and biological features of these models have been reviewed [24]. The majority of these models were generated using promoters such as MMTV (and others) to overexpress genes known to promote tumorigenesis including *myc*, *ras*, *fgf3*, *tgfa*, and *wnt1*. Here, we will focus on the most commonly used and relevant models that are considered to closely mimic human DCIS progression. One of the earliest and well-established models of mammary tumorigenesis is the MMTV-PyMT (MMTV-PyVT) transgenic mouse, in which polyomavirus middle T antigen is driven by MMTV-LTR [41]. Mammary lesions in these mice progress through four distinct stages: atypical hyperplasia, adenoma (MIN), early carcinoma and late carcinoma [42]. These mice rapidly develop multifocal mammary adenocarcinomas by 5 weeks of age, and pulmonary metastasis occurs in >90% of females. While progression through MIN is an advantage and could arguably represent the transition from DCIS to invasive cancer, the robust and rapid development of multifocal primary tumors at a young age presents challenges to studying the slow progression of DCIS, and thus is primarily used to study metastasis. To overcome this limitation, MacLeod and colleagues developed MIN outgrowth lines (MINO) by syngeneic serial transplantation of pre-invasive MIN lesions from MMTV-PyMT mice [43–45]. Six MINO lines were established with distinct morphological patterns of differentiation and varying latencies and metastatic potential. The phenotypes were stable over multiple transplant generations and gene expression profiling showed that premalignant stages of each line shared characteristics with human DCIS, as well each corresponding tumor. Each line met the test-by-transplantation criteria, and a number of chemoprevention studies have been performed, suggesting that the MINO model may accurately model human DCIS progression [46, 47].

The receptor tyrosine kinase ErbB2 (HER2) is amplified in about 25% of human breast cancers and is associated with an aggressive phenotype and reduced response to hormone therapies. As such, a number of ErbB2 transgenic mouse models have been developed in which human *erbb2* or wildtype or activated *neu* (the rat homologue) is expressed under the control of the MMTV promoter [reviewed in [48, 49]]. Although variants of the MMTV-Neu models display different tumor latencies (3–6 months), likely due to differences in transgene expression and integration sites, mammary lesions develop atypical hyperplasias and focal or multifocal tumors with varying degrees of lung metastasis. The hyperplasias that develop in various ErbB2 transgenic models have been shown to share common cytological features with human DCIS [50]. Similar to the MMTV-PyMT model, ErbB2-induced mammary lesions are morphologically lobular (HAN) [41, 51, 52], reminiscent of early histological changes in human terminal ductal lobular units (TDLUs) and HELUs. Despite sharing these characteristics with preneoplastic lesions, there are notable differences in ErbB2 GEMMs as compared to human disease [reviewed in [53]]. Genetic alterations in *erbb2* occur frequently in high grade DCIS [54], but whether the progression of MMTV-Neu-induced hyperplasias accurately mimics human HER2^+^ DCIS progression has yet to be determined. Nonetheless, ErbB2 mouse models have proved useful for understanding *erbb2* genetics and HER2 signaling in human breast cancer.

Welm and colleagues developed a unique transgenic model of early stage progression using an inducible dimerization system under the control of the MMTV promoter to activate fibroblast growth factor receptor (FGFR) 1 [55]. Treatment with the drug AP20187 (or B/B) causes receptor dimerization and consequent phosphorylation and activation in the mammary epithelium. Three distinct types of lesions were observed in FGFR1-activated mammary glands after short-term treatment, and chronic treatment of mice induced invasive tumors. Type I lesions appeared as early as three days after treatment and were characterized by lateral alveolar buds associated with the primary ducts with an intact basement membrane, reminiscent of pregnancy-induced lobuloalveoli. By two weeks post-treatment, type II lesions formed consisting of multi-layered epithelium and collapsed lumens, but often an intact basement membrane. Type III multifocal lesions appeared 4 weeks after FGFR1 activation and were multicellular, well-vascularized and invasive. Lesions induced by FGFR1 activation likely represent HAN, as lateral budding occurs outside of the ducts. Although these lesions contain a mixture of PR^+^ and PR^−^ cells, they exhibit hormone-independence, similar to most MMTV-driven models. The predictable timecourse for the development of three distinct types of early lesions allows for unique opportunities to study the progression from premalignant to invasive cancer. As such, this mouse model has proven useful for the study of FGFR1-driven tumorigenesis [56–58].

SV40 T antigen has also been used to generate mouse models of tumor progression, including the well-characterized C(3)1Tag mouse model that exploits the hormonally regulated rat prostate steroid binding protein (PSBP) C3 promoter. Expression of T antigen results in the targeted inactivation of p53 and Rb, leading to the development of prostate tumors in males and mammary tumors is females. Similar to MMTV-PyMT and iFGFR1 mice, mammary lesions progress through distinct premalignant/pre-invasive stages in a predictable timecourse, an attractive feature to allow for the study of early stage progression. Female mice initially show normal ductal development, progress to ductal atypia by 6–8 weeks of age (low grade MIN), high grade MIN (similar to DCIS) by 12–14 weeks, and invasive cancer by 16 weeks or later [38]. Although less studied, lesions generated by T antigen expressed by the whey acidic protein (WAP) promoter (WAP-T mice) also progress through distinct premalignant stages and show remarkable histological similarities to human DCIS [40]. Unlike MMTV-PyMT- and MMTV-Neu-induced lesions, SV40 T antigen-driven models appear to represent DH rather than HAN, forming solid nests of poorly differentiated cells that may originate within the terminal ducts of the mammary gland, where the transgene is expressed [50, 59]. Green and colleagues used comparative transcriptomics (microarray and laser capture microcopy) to analyze gene expression in pre-invasive MIN and invasive tumors, and showed that gene expression profiles were similar [59]. These findings are corroborated by early gene expression profiling of human breast lesions from ADH, DCIS and IDC in which the majority of genetic alterations important for tumor progression occurred by the ADH stage, and persisted throughout progression [60]. Transcriptomic profiling of tumors from T antigen-driven models show that they most closely represent basal-like breast cancers [61], however, a thorough genetic and molecular analysis of the different pre-invasive stages has not been performed.

**Fig. 1:**
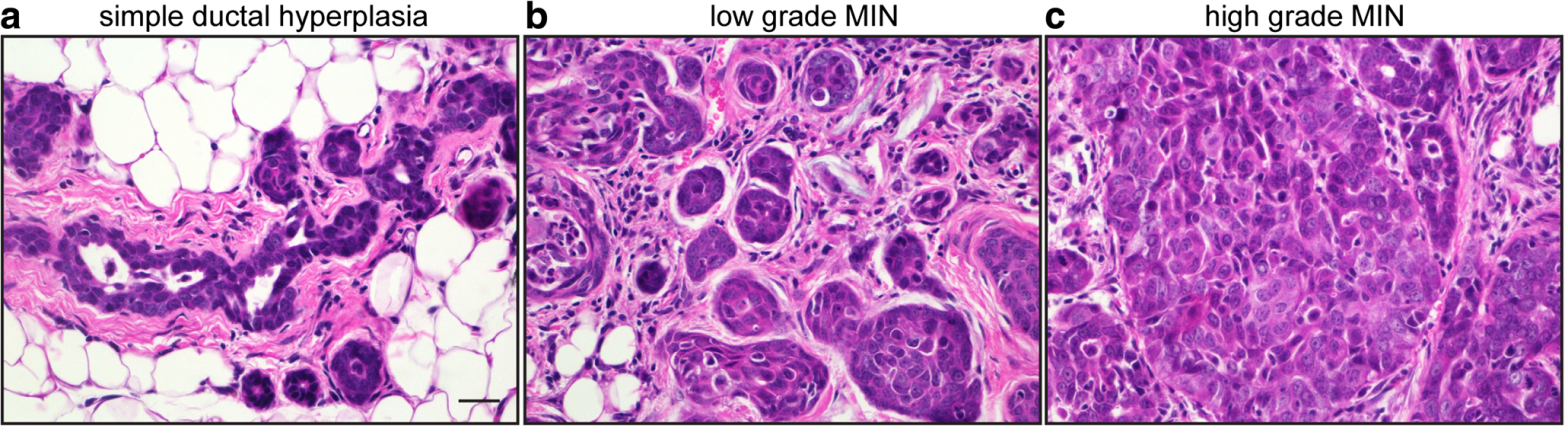
Histopathology of the PN1a p53^−/−^ outgrowth line at three distinct stages of progression. **a** Simple ductal hyperplasia with empty lumens, (**b**) low grade MIN showing multilayered epithelium and collapsed lumens, (**c**) high grade MIN with multicellular, multifocal lesions and areas of invasion. Scale bar: 10 μm

*TP53* is the most frequently mutated gene in breast cancer, with genetic alterations in about 30% of breast cancers, predominantly of the basal subtype. A number of *TP53*-null GEMMs or models of somatic loss of *TP53* have been generated to mimic *TP53* alterations in human breast cancer. Medina and colleagues developed and characterized premalignant outgrowth lines derived from p53^−/−^ (or p53^±^ ) Balb/c mammary glands [62, 63]. These transplantable lines may be histologically ductal or alveolar, and progress from simple ductal hyperplasia to DCIS (cribriform or comedo) (Fig. 1). Fourteen premalignant outgrowth lines were established [termed PH (p53^±^ ) or PN (p53^−/−^)] and show notable differences in ER expression, aneuploidy, tumor latency and the ability to progress to invasive cancer [33]. Differences in tumor forming potential allows for unique opportunities to understand why some pre-invasive lesions progress to invasive cancer while others remain indolent [64], a major barrier in the field of DCIS biology. Another important feature of this model is that many of the outgrowth lines are ER^+^ at the ductal hyperplasia stage but give rise to ER^−^ tumors. Similarly, C(3)1Tag and WAP-T mice form DH and are initially ER^+^ followed by the progressive loss of ER at the DCIS stage. In human breast cancer, low grade DCIS tends to be ER^+^, while high grade DCIS is associated with loss of ER/PR expression and frequent alterations in p53. Unlike lesions induced by chemical carcinogens or in C(3)1Tag mice, the p53^*−/−*^ premalignant lesions are ovarian hormone-dependent, a characteristic unique to this model [62, 65]. These data suggest that the p53^−/−^ premalignant outgrowth lines may most accurately mimic human DCIS progression.

It should be noted that in addition to GEMMs, early breast cancer progression has been modeled in the rat. Li and colleagues showed that prolonged exposure of estrogen (17β-estradiol) induces focal dysplasias and DCIS-like lesions that progress to tumors in August Copenhagen Irish (ACI) rats [66]. As compared to rats treated with chemical carcinogens that induced diploid tumors, estrogen-induced tumors were primarily aneuploidy, which closely resembles human DCIS and invasive breast cancer. As these are ER^+^ hormone-dependent lesions, estrogen-treated ACI rats may arguably represent the most accurate animal model for studying DCIS progression and spontaneous breast cancer [67, 68].

In conclusion, mouse models of MIN have proved useful tools that show morphological similarities to premalignant lesions in humans. Despite their utility, the mechanisms that mediate the transition from pre-invasive to invasive cancer remain largely unknown. In DCIS, pre-invasive cells are confined by an intact myoepithelium and basement membrane, which is disrupted during the switch to invasive cancer. It is unclear as to whether (1) the myoepithelium is first disrupted, allowing DCIS cells to invade the stroma, or (2) DCIS cells acquire the ability to invade the basement membrane, consequently disrupting the myopeithlium. A major limitation of GEMMs of early stage progression is that most models do not recapitulate localized invasion. Studies using the iFGFR1 mice [55], WAP-T mice [40]and the p53^−/−^ outgrowth lines [64] have shown disruption of the basement membrane during the transition to invasive cancer, which may be indicative of localized invasion of DCIS, overcoming this limitation. Another caveat is that nearly 100% of GEMM-induced MIN will progress to invasive cancer. One exception are the p53^−/−^ outgrowth lines that have varying abilities to form invasive tumors. In human breast premalignancy, ~5–10% of patients with ADH and ~50% of patients with DCIS have a high risk of developing invasive cancer [69]. Developing and utilizing models that more accurately mimic this prognostic heterogeneity as well as hormone dependence (discussed above) will be critical for identifying DCIS patients at risk for developing invasive cancer.

## XENOGRAFT Models of Human Ductal Carcinoma In Situ

Early attempts at developing human xenograft models of premalignant breast lesions date back to 1975 when Outzen and Custer transplanted small fragments of human breast hyperplasia into cleared mammary fat pads of nude mice [70]. The xenografted tissue proliferated and maintained a similar histology in vivo as the original patient’s biopsy [71]. More recently, Warnberg and colleagues implanted human DCIS tissue fragments subcutaneously in nude mice to study the therapeutic efficacy of a farnesyl transferase inhibitor. The take rate for the DCIS xenografts was about 66% and DCIS lesions were maintained in mice for up to 21 days [72]. Additionally, Espina V, et al. demonstrated successful xenotransplantation of freshly procured DCIS organoids and propagated DCIS spheroids in vitro from biopsy or surgical specimens. This group reported that invasive tumors formed at a rate of ~80% (21/27 cases transplanted) from both freshly procured as well as in vitro propagated organoids [73] .

With the notion that human DCIS initiates inside the ducts, Behbod and colleagues, developed the mouse-intraductal (MIND) model. MIND involves intraductal injection of DCIS cell lines and patient-derived DCIS epithelial cells into the primary mammary ducts of immunocompromised mice [74]. Similar to human DCIS, intraductally injected DCIS epithelial cells form in situ lesions followed by invasion into the surrounding stroma as cancer cells bypass the natural barriers of the myoepithelial cell layer and basement membrane [74–76]. Initially, they utilized MCF10DCIS (herein referred to as DCIS.COM), SUM225CWN (herein referred to as SUM225) cell lines and one case of a patient-derived DCIS [74]. The DCIS-like lesions generated from these cell lines form in situ lesions surrounded by the mouse myoepithelial layer as early as two weeks after injection and slowly progress to invasive lesions by 8–10 weeks [74]. DCIS-like lesions formed by the DCIS.COM cell line lack expression of ER, PR and HER2, while DCIS-like lesions generated by SUM225 are HER2-positive and lack expression of ER and PR.

**Fig. 2:**
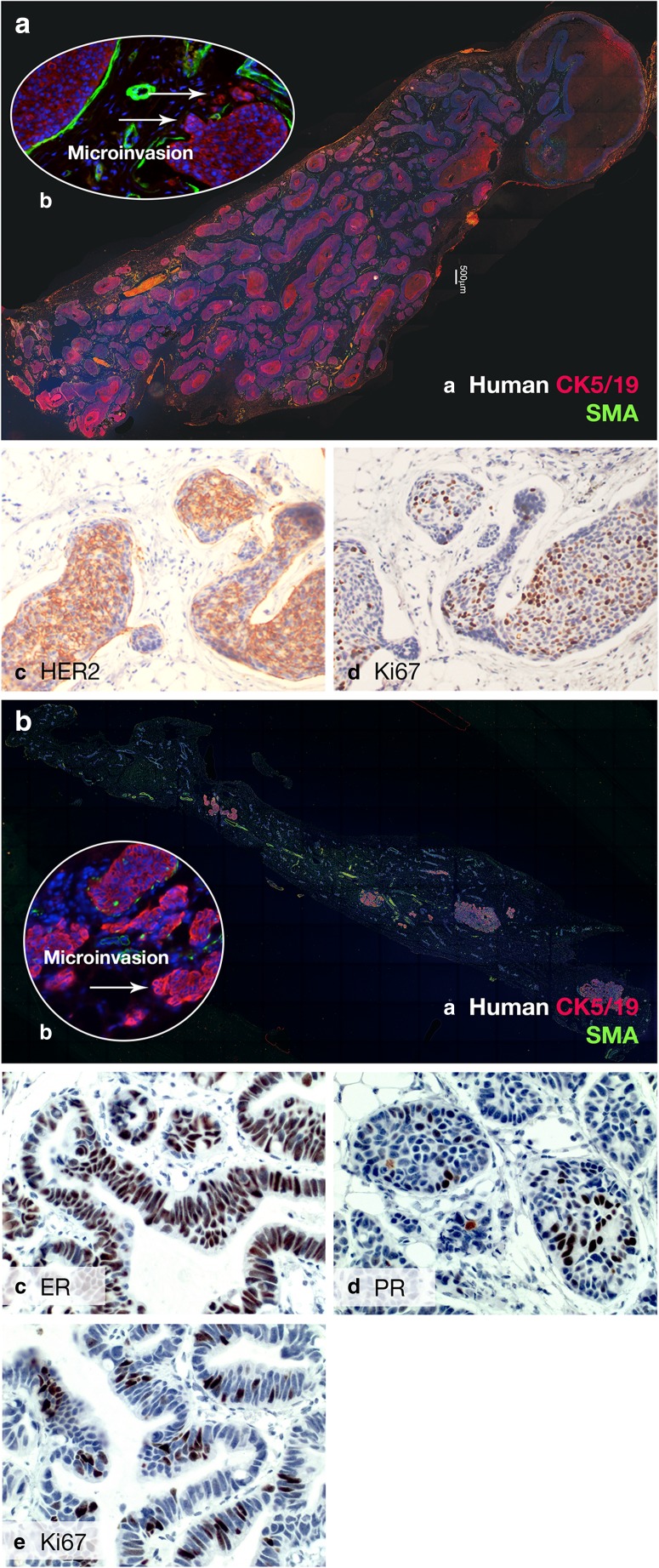
Patient-derived DCIS MIND xenograft models. **a** Immunofluorescence (IF) image from a section of a MIND xenograft generated by intraductal injection of epithelial cells derived from a patient DCIS that was ER/PR-negative, Ki67 25%; nuclear grade 2–3, comedo and solid type 12 months post-transplant. *a* IF using anti-human CK5/19 and SMA. *b* An area of microinvasion shown by the white arrow. *c* HER2 immunohistochemistry (IHC). *d* Ki67 IHC. **b** IF image of a section of a MIND xenograft 12 months following intraductal transplantation. Patient DCIS was ER/PR positive, Ki67 10%; nuclear grade 2–3, comedo and solid type. *a* IF using anti-human CK5/19 and SMA. *b* An area of microinvasion shown by the white arrow. *c* ER IHC. *d* PR IHC. *e* Ki67 IHC

In 2011, Valdez and colleagues reported that the MIND model also supported the reproducible growth of patient-derived DCIS in NOD-SCID IL2rγ (NSG) mice [75]. A fraction of patient- derived DCIS MIND xenografts show invasive progression at a rate of ~40% upon long term follow up of 6–12 months (Behbod, F unpublished results). The overall xenograft take rate is ~70% (~ 110/164 xenografts). The MIND model supports intraductal growth of epithelial cells derived from a variety of human premalignant and malignant lesions including hyperplasias, subtypes of DCIS, subtypes of invasive ductal carcinoma and normal mammary epithelial cells from BRCA mutation carriers (Behbod, F unpublished results). Patient-derived DCIS MIND xenografts, similar to patient DCIS, retain expression of ER, PR, HER2 and Ki67 (Fig. 2). Retention of ER and PR expression is a remarkable advantage of MIND over standard cleared mammary fat pad transplantation methods. A side-by-side comparison of patient-derived xenografts generated by MIND as compared to the standard cleared fat pad transplantation showed that the MIND method is superior for modeling ER^+^ invasive breast cancers. ER^+^ MIND xenografts generated from invasive ER^+^ patient tumor cells also more closely resembled their clinical counterparts with respect to histology and tumor kinetics including proliferation index and presence of key radiologic features such as microcalcifications. Most importantly, ER^+^ MIND xenografts metastasized to the same sites as their clinical counterparts, including bone and brain, but rarely to liver or lungs, whereas xenografts generated by the standard fat pad transplantation metastasized to lungs, less frequently to brain and none to bone [77].

The DCIS cell line and primary DCIS MIND models are valuable tools for studying temporal molecular changes associated with DCIS non-invasive to invasive transition. For example, Elsarraj H.S., et al., studied gene expression changes in DCIS.COM and SUM225 DCIS MIND models during a transition from non-invasive to invasive transition, at 2, 6 and 10 weeks [78]. These time points were selected in order to accurately reflect the molecular changes, as the DCIS lesions were formed between 2 and 6 weeks and progressed past the myoepithelial layer and the basement membrane by 10 weeks. They found a significant upregulation in genes belonging to the canonical Wnt, STAT3 and EMT pathways. They validated the role of B cell lymphoma-9, a cofactor in the canonical Wnt signaling, in promotion of DCIS invasive progression by knockdown studies in DCIS cell line models, DCIS.COM and SUM225. Interestingly, subsequent studies found BCL9 to promote simultaneous co-activation of both the canonical Wnt and STAT3 pathways to drive EMT and DCIS invasive progression (unpublished results).

Other investigators, by utilizing the DCIS cell line MIND models, validated the role of suppressors of DCIS progression. Lee, S., and colleagues [79] performed laser capture microdissection of pure DCIS and DCIS with associated IDC followed by Affymetrix microarray gene profiling. This study found 470 differentially expressed genes, 74 of which showed an overlap with 2 or more of 9 other similar studies. The 74-gene profile was able to correctly categorize 85 to 96% of the samples in their study as well as two similar independent studies, respectively. They selected four genes to validate using knockdown strategy in three DCIS cell line MIND models, DCIS.COM, SUM225 and DCIS.01. Progression to IDC was significantly increased by suppressing four genes, CSTA (a protease inhibitor), FAT1, DST and TMEM45A (genes involved in cell adhesion and signaling). Thus, their study validated the role of four suppressor of DCIS invasive progression using DCIS cell line MIND models.

Other investigators have utilized xenograft models of DCIS.COM to study the cellular and molecular mechanisms of DCIS invasive progression. Using the DCIS.COM MIND model, Russell and colleagues reported that the DCIS non-invasive to invasive transition was associated with the progressive loss of myoepithelial p63, followed by calponin and finally α-smooth muscle actin (SMA) [80]. Hu and colleagues analyzed the contribution of myoepithelial cells and fibroblasts to the progression of DCIS to invasive carcinoma using subcutaneous transplantation of DCIS.COM. This group showed that progression to invasion was inhibited by normal myoepithelial cells and promoted by fibroblasts derived from invasive carcinomas and rheumatoid arthritis. By detailed molecular and histological analysis, this group demonstrated that DCIS.COM subcutaneous xenografts were similar to human high grade comedo DCIS. The xenografted lesions were surrounded by a basement membrane, positive for laminin 5 and contained a layer of cells positive for the myoepithelial markers SMA, CD10 and p63. They also confirmed that DCIS.COM cells possessed progenitor cell properties giving rise to both luminal and myoepithelial cells upon transplantation. Molecular analysis of DCIS.COM-derived myoepithelial-specific (integrin β6^+^) and luminal specific (MUC1^+^) cells led to the discovery that a complex interaction between epithelial, myoepithelial and stromal signaling pathways including TGFβ, hedgehog, cell adhesion and p63 were required for the loss of myoepithelial cells in DCIS and progression to invasion [81]. As such, subcutaneous transplantation of DCIS.COM may serve as another useful tool for characterization of the molecular and cellular processes underlying DCIS invasive progression.

## Conclusions and Future Directions

Human DCIS, similar to invasive breast cancer, is a multifaceted disease characterized by inter- and intra-tumoral heterogeneity, diverse subtypes, and increased genomic alterations over time. Despite advances in modeling DCIS in mice, human DCIS continues to be treated with a “one-size fits all” approach, where all DCIS patients receive surgery and/or radiation therapy. As such, it remains unclear as to why some patients progress to invasive disease, while others remain benign. Thus, it is crucial that adequate models that mirror human breast premalignancy should continue to be developed and utilized.

The availability of a suitable model would enable the identification of molecular events important for the transition to invasive cancer by comparing DCIS lesions that progress to invasive cancer compared to those that remain non-invasive. This approach has been used with p53-null outgrowth lines [64]. However, this strategy assumes that human DCIS evolution in mice (mouse models and patient-derived xenografts) are similar to that in humans. Developing humanized MIND xenograft models may help reduce the differences between the human and mouse tumor microenvironment, providing a more relevant model to understand the complex interactions between the stroma and pre-invasive lesions. In summary, combining appropriate GEMM models of early progression with current xenograft models, such as MIND, with humanized stroma will be critical for understanding molecular mechanisms of localized DCIS invasive progression.

## Abbreviations

DCIS: Ductal carcinoma in situ
ADH: Atypical ductal hyperplasia
IDC: Invasive ductal carcinoma
ALH: Atypical lobular hyperplasia
LCIS: Lobular carcinoma in situ
HER2: Human epidermal growth factor receptor 2
ER: Estrogen receptor
PR: Progesterone receptor
HAN: Hyperplastic alveolar nodules
MIN: Mammary intraepithelial neoplasia
GEMM: Genetically engineered mouse models
MMTV: Mouse mammary tumor virus
DH: Ductal hyperplasia
HELU: Hyperplastic enlarged lobular units
MMTV-LTR: Mouse mammary tumor virus-long terminal repeat
PyMT: Polyomavirus middle T antigen
MINO MIN: Outgrowth line
TDLU: Terminal ductal lobular unit
FGFR1: Fibroblast growth factor receptor 1
SV40: Simian virus 40
PSBP: Prostate steroid binding protein
iFGFR: Inducible fibroblast growth factor receptor
WAP-T: Whey acidic protein-T antigen
ACI: August Copenhagen Irish
MIND: Mouse-intraductal
NSG: NOD-SCID IL2rγ
BRCA: Breast cancer susceptibiltiy gene
BCL9: B cell lymphoma 9
EMT: Epithelial mesenchymal transition
SMA: α smooth muscle actin
MUC1: Mucin 1
STAT3: Signal trnasducer and activator of transcription 3
